# Fabrication, characterization and antifungal evaluation of polyphenolic extract activated keratin starch coating on infected tomato fruits

**DOI:** 10.1038/s41598-022-07972-0

**Published:** 2022-03-14

**Authors:** Olarewaju M. Oluba, Onome Obokare, Opeyemi A. Bayo-Olorunmeke, Samuel I. Ojeaburu, Olayemi M. Ogunlowo, Emenike O. Irokanulo, Oghenerobor B. Akpor

**Affiliations:** 1 Food Safety and Toxicology Research Unit, Department of Biochemistry, College of Pure and Applied Sciences, Landmark University, Omu-Aran, Kwara State Nigeria; 2 Department of Zoology, University of Ibadan, Ibadan, Oyo State Nigeria; 3 Department of Biochemistry, Faculty of Life Sciences, University of Benin, Benin-City, Edo State Nigeria; 4 Department of Biochemistry, Federal University of Technology Akure, Akure, Ondo State Nigeria; 5 Department of Food Science and Microbiology, College of Pure and Applied Sciences, Landmark University, Omu-Aran, Kwara State Nigeria; 6 Department of Microbiology, Afe Babalola University, Ado Ekiti, Ekiti State Nigeria

**Keywords:** Biochemistry, Biological techniques, Biotechnology

## Abstract

In recent times, the application of protein-based bio-composite edible films in postharvest preservation of food and agricultural products is attracting increased attention due to their biodegradability, eco-friendliness and sustainability. In this study, an avocado pear peel polyphenolic extract enriched keratin-starch composite film was fabricated, characterized and evaluated for antimicrobial activity against fungal infected tomato fruits after 6 days of storage at room (25 ± 2 °C) temperature. The SEM/EDX and FTIR results revealed the successful film formation with high degree of compatibility and homogeneity. Following a 6-day post-coating loss in weight of the coated tomato fruits decreased significantly (*p* < 0.05) with increasing extract concentration while titratable acidity showed a significant (*p* < 0.05) increase with increasing extract load. Ascorbic acid and lycopene contents were significantly (*p* < 0.05) higher in the avocado pear peel polyphenolic extract-loaded films. No significant effect was observed in catechol oxidase activity of the tomato extract across the different treatment groups. In addition, fungal growth inhibition showed a dose dependent increase consistent with avocado pear peel polyphenolic load in coated tomato fruits compared to control. Results obtained in this study showed that polyphenolic activated keratin-starch coating was able to reduce spoilage-induce weight loss as well as conserve the overall quality (including titratable acid levels, lycopene and ascorbic acid contents) of fungal-infected tomato fruit and reduce microbial growth. Therefore polyphenolic activated keratin-starch coating could serve as a sustainable and ecofriendly postharvest preservation method to prolong the shelf life of tomato fruits.

## Introduction

The global population is projected to witness an astronomical growth in the next few years. This projection is worrisome especially if not matched with proportionate food production. This is a major challenge especially in developing countries where an estimated 1.2 billion people go to bed hungry^1,2^. One major strategy to ensure food security is to reduce wastage due to food spoilage and deterioration. Thus, research focused on increasing shelf life, as well as ensuring microbial safety of fresh fruits and vegetables through edible coating technology is an effort in the right direction.

Edible coatings are solutions applied to fresh fruits and vegetable in order to maintain their freshness as well as prevent deterioration that may be due to dehydration, loss of appearance, flavor and nutritional qualities of such produce within the time taken for them to reach the consumer. Huge losses in terms of quantity and quality are recorded during postharvest handling of food and agricultural products. Majority of these losses are attributed to poor and/or ineffective postharvest preservation and storage management systems. The widely use of low temperature (4–8 °C), especially in the preservation of lightly foods, though effective in inhibiting undesirable enzyme activities most, often results in increased rate of respiration, as well as ethylene production^3^. In addition, some cold-tolerant pathogenic organisms thrive well under refrigeration condition^4^. Over the past 50 years, research efforts have focused on the encasement of food product with organically derived films and coatings in order to retard or maintain the migration of molecules and enzymes involved in their deterioration at natural level.

The shift for a sustainable eco-friendly alternative for hydrocarbon-based polymeric materials in recent times has led to a renewed interest in the fabrication of edible films and coatings from agricultural wastes. In this regard, attempts have been made to fabricate films and coatings from renewable resources, such as proteins and polysaccharides. The application of plant and animal wastes as protein sources for coatings production opens a new channel of value addition in the agricultural chain, hence, improving the economics of food processes. Protein-based coatings remain a sought after due to their potential ability to form films offering barrier to both water and oxygen^5,6^.

Keratin, a fibrous protein, has been recommended as excellent starting materials for films and coatings due to its disulphide bonds, which when reduced facilitate intermolecular crosslinking of protein chains. The presence of charged groups resulting from polar amino acids in the keratin polypeptide chain generates a chemical gradient. This chemical potential aids in the stabilization of keratin films via various intermolecular forces in hydrogen bonding and electrostatic interaction, as well as disulfide bridges^7^. In addition, the semi-crystalline nature of feather keratin contributes to its extraordinary strength and toughness^8^. Based on the report of Oluba et al*.*^9^ ginger is a non-conventional source of starch for industrial applications. Ginger starch was reported to be made up of 39.1% amylose content, and with potential desirable attributes for film formation including 1.87% optimum solubility value at 90 °C, swelling power of 132.2% and gelatinization temperature of 65.7 °C.

Coating layered on the surfaces of fruits offer barriers to both moisture and gases in the processing, preservation and storage of foods and agricultural products^10^. The use of coating for protection purposes represents an economical advantage, thus avoiding the need for climate-controlled storage, which incurs operational costs and requires special equipment^11^. Biocomposites fabricated from a combination of starch and keratin could be applied as a protective layer over the surface of fruits so as to modulate their internal physiology. Starch due to its hydrophilic nature offer poor resistance to water but exhibits excellent gas barrier property^12^. Keratin on the other hand, offers suitable barrier property against moisture and gases under low relative humidity^12^. Thus, keratin-starch bio-composite film offers excellent barriers to moisture and gases as opposed to film fabricated from keratin only or starch only. In addition, keratin-starch films could also serve as vehicles to introduce nutraceutical products with antimicrobial activity^13^.Therefore, a combination of keratin and starch in the fabrication of a biocomposite film as hypothesized in this study is desirable.

Avocado peel is a major waste generated in avocado pear processing industries. An estimated 35–50% waste^14^ is produced from the avocado pear processing plant with avocado peel constituting a substantial 20–25%. However, given its rich content of polyphenolics, avocado peel is considered a sustainable, low cost biomass for reclaiming phenolic compounds from agro-wastes. Polyphenolics extracted from avocado peel has exploited in many industries including pharmaceuticals, cosmetics and beauty as well as food preservation^15^ as an antimicrobial and antioxidant agent.

An estimated 30% of harvested tomato fruits are lost due to microbial spoilage during post-harvest handling^16^. Microbial spoilage of tomato fruit may occur at any stage during the growing season, harvesting, handling, transport and post-harvest storage. Fungi including *Aspergillus niger, Aspergillus flavus* and *Penicillium notatum* have been identified as the major causative agents of tomato spoilage in Nigeria^16^. Fungal fruit rots are the most prevalent form of tomato spoilage. The fungal rots thus constitute a serious production problems and become menace for successful cultivation of tomatoes worldwide. The principal fungal fruit rots reported globally with varying intensities on tomato includes Alternaria rot caused by *A. solani* and *A. tenuis*, Phytophthora rot caused by *P. infestans*, *P. nicotianae* var. parasitica, Anthracnose ripe rot caused by *Colletotrichum phomoides*, Phoma rot *P. destructiva* and Fusarium rot caused by *Fusarium spp*.^17,18^. The successful fabrication of a keratin-starch composite film with desirable attributes for food packaging by Oluba et al.^9^ warrant further study aimed at improving its functionality with regard to food packaging and preservation. Therefore, the present study was aimed at the functionalization of keratin-starch composite with polyphenolics extracted from avocado pear peel and to evaluate its antifungal activity against fungi-infected tomato fruits when applied as a coating.

## Materials and methods

### Ethics approval

This study does not require any formal consent as it does not include any human participation or animal experimentation. All the experiments were carried out in accordance with relevant institutional, national, and international guidelines/legislation.

### Chemicals and reagents

Analytical grade chemicals purchased from Sigma-Aldrich Ltd (UK) were used for the study.

#### Plant materials

Fresh ginger rhizomes, matured green avocado pear (*Persea americana*) and fresh tomato fruits of similar size, shape and colour were purchased from the main market in Omu-Aran, Kwara State, Nigeria and transported to the Biochemistry Laboratory, Landmark University, Omu-Ara, Kwara State, Nigeria. Identification and authentication of the plant samples were carried at the Department of Plant Science Herbarium, University of Ilorin, Ilorin, Nigeria where the respective vouchers were deposited.

### Microwave assisted polyphenol extraction

Total polyphenol extract was carried out using the modified microwave assisted method of Kumar et al.^19^ with little modifications. Powdered avocado pear peel (50 g) was put in 500 mL Erlenmayer flask containing 250 mL ethanol (50%). The Erlenmayer flask with its content was then put inside a BP090 microwave oven (Microwave Research & Applications, Inc. Illinois, USA). The flask was securely connected to a vertical condenser. The extraction process was performed at 300 W for 5 min. Thereafter, the liquid extract was separated from the residue through vacuum filtration. The liquid extract was vacuum evaporated at 40 °C to obtain the concentrated polyphenol extract.

### HPLC analysis

The characterization of the avocado peel polyphenolic extract was carried out according to Šeruga et al.^20^ method.

### Starch extraction

The process undergone in the extraction of starch from ginger rhizome was as detailed by Oluba et al.^9^. Thoroughly washed peeled ginger rhizomes soaked in sodium metabisulphite solution (1%) and blended into fine paste with electric blender. The ginger starch paste was dispersed into a large volume of water, stirred vigorously and left to stand for 12 h. The water was then carefully poured off while the starch paste was scraped into petri dish, oven-dried at 30 °C to constant weight, weighed and stored at 4 °C.

### Chicken feather

White coloured waste feathers were collected from the slaughter unit of Landmark Commercial Farms, Landmark University, Omu-Aran, Nigeria.

### Keratin extraction

The extraction of keratin from chicken feather waste followed the procedure detailed by Oluba et al.^9^.

### Preparation of active keratin-starch coating

The active keratin-starch edible coating was prepared according to the procedure described by Oluba et al*.*^9^ with little modifications. A 5% starch solution was prepared and gelatinized by heating on a laboratory hot plate at 70 °C with constant stirring. Keratin solution was made by dissolving 5 g of the extracted dried keratin powder in 100 mL of 0.1 M NaOH and heated on a laboratory hot plate at 70 oC with constant stirring while adding 10 mL of 3% sodium sulphite to prevent the realignment of the di-sulphide bonds. Different blends of starch, keratin and avocado peel polyphenolic extract were prepared by varying the concentration of extract added (Table 1). To each starch-keratin blend, 2 mL of glycerol was added as plasticizer after which the mixture was heated at 100 °C on a laboratory hot plate, with constant stirring. The prepared active coating was divided into two portions. The first portion was poured into a glass petri dish and oven-dried at 75 °C for 72 h, this was used for the characterization analyses. The second portion was used for the coating experiment.

**Table 1:** Experimental design.

Group	Treatment
CTR	Infected tomato coated with distilled water
K-SC	Infected tomato coated with keratin-starch film only
K-SC + 0.2	Infected tomato coated with keratin-starch film containing 0.2 mL avocado peel polyphenolic extract
K-SC + 0.4	Infected tomato coated with keratin-starch film containing 0.4 mL avocado peel polyphenolic extract
K-SC + 0.6	Infected tomato coated with keratin-starch film containing 0.6 mL avocado peel polyphenolic extract
K-SC + 0.8	Infected tomato coated with keratin-starch film containing 0.8 mL avocado peel polyphenolic extract
K-SC + 1.0	Infected tomato coated with keratin-starch film containing 1.0 mL avocado peel polyphenolic extract

### Fungi isolation

One gram of spoilt tomatoes was cut with a sterile scalpel and submerged in 10 mL Sabouraud dextrose broth (SDB) for 72 h. One milliliter (1 mL) of the resulting broth containing fungal growth was plated in sterile Sabouraud dextrose agar (SDA) plates, using the standard pour plating technique. To inhibit bacterial growth, ciprofloxacin antibiotic was added to the sterile SDA (cooled to 40 °C) at concentration of 50 mg/L. Before por plating, the sterile SDA was first cooled to 40 °C. The plates were incubated at 25 °C for 72 h and observed for growth. The resulting distinct colonies were further subcultured in fresh sterile SDA plates and incubated to obtain pure cultures. The pure cultures were tentatively identified using cultural and morphological features such as colony growth pattern, and conidial morphology as *A. flavus* and *A. niger* and were then used to infect fresh tomatoes^21^.

### Fruit inoculation and coating experiment

Two hundred and ten (210) freshly purchased matured tomato fruits of similar size and shape, free from lesions and postharvest diseases were washed with 2% hypochlorite solution (^v^/_v_) and distilled water and allowed to air dry at room temperature (25 ± 2 °C). The tomatoes were then divided into seven groups of ten tomatoes each (each treatment group was replicated three times). Tomatoes were superficially wounded once in the equator with a sterile scalpel with a probe tip 1 mm wide and 2 mm in length. The wound was then inoculated with 10 μL suspension of a mixture of *A. flavus* and *A. niger* containing about 1 × 10^6^ spores/mL in sterile distilled water. The infected tomato fruits were then incubated at 20 °C for 24 h to resemble common fungal infections before the coating experiment^22^. Following the incubation period, inoculated tomato fruits were randomly assigned to seven treatment groups (Table 1) and coated with their respective coating film by immersion for 30 s. Each treatment had three replica of 10 tomato fruits each. Thereafter, the coated fruits were drained, and left to air-dry at room temperature (25 ± 2 °C) for 12 h before being placed on plastic trays on corrugated cartons to avoid contact and then stored for 6 days at room temperature (25 ± 2 °C) under laboratory conditions.

### Characterization of the fabricated polyphenol extract-activated film

Surface and internal morphology as well as elemental composition of the fabricated films were determined using scanning electron microscopy/electron dispersion X-ray (SEM/EDX). Quantitative determination of surface functional groups of the film was carried out using Fourier Transform Infra-red (FTIR) Spectroscopy. The transparency of the film was measured according to Santacruz et al.^23^ using a UV–VIS spectrophotometer (Jenway 7305, UK) at 560 nm.

### Water solubility determination of the fabricated polyphenol extract-activated film

The solubility of the fabricated avocado pear peel polyphenolic extract-based keratin-starch film solubility in water was determined according to Fakhouri et al*.*^24^ method with slight modification. A given portion of the film was cut, weighed and dried at 75 °C for 24 h after which it was immersed in a beaker containing 50 mL of water, and stirred continuously for 24 h. The biofilm sample was then removed and dried again at 75 °C for 24 h. The solubility (expressed in %) was calculated as the difference in weight before and after immersion. Determination of solubility of samples in acidic medium was carried out by the same process with a difference in the immersion solution which for acidic medium is 1 M hydrochloric acid.

### Physicochemical characteristics assessment of tomato fruits after the coating experiment

Following a six-day post coating period, tomato fruits from each treatment group was homogenized and filtered using Whatman™ number 1. The filtrate was stored in plain sterile bottles and kept at 4 °C until required for further analysis.

### pH determination

Ten milliliter (10 mL) filtrate was diluted with 50 mL distilled water and the pH of the resulting solution determined using an electronic pH meter (H12210 pH meter).

### Titratable acidity

Five milliliter (5 mL) of filtrate was diluted with 20 mL distilled water. Three drops of phenolphthalein was added as an indicator to the solution. The solution was titrated against 0.05 M sodium hydroxide till a persistent pink colour was formed. Values were expressed as g lactic acid/100 g of sample^25^. Calculated as:

$$ TA = \frac{M\,{\rm NaOH} \times {\rm mL\;NaOH} \times 0.09 \times 100}{{mL\;of\; sample}} $$

### Ascorbic acid assay

The 2, 6 dichlorophenol indophenol assay titrimetric method was used for the determination of ascorbic acid. d. The dye solution was standardized by pipetting 5 mL of standard ascorbic acid solution into a 100 mL conical flask and titrated against 2, 6 dichlorophenol indophenol dye solution till a persistent light pink color appeared. The volume of the dye used was recorded as $V_{1}$. Thereafter, 5 mL of sample was diluted three-folds in a 100 mL volumetric flask with metaphosphoric acid. The diluted sample solution (10 mL) was pipetted into a conical flask and titrated against the dye till a light pink color appears which persists for 30 s ($V_{2}$).

The ascorbic acid content was calculated using the equation:

$$ Ascorbic\; acid\; content = \frac{{0.5\;{\text{mg}} \times V2_{2} \times 100\;{\text{mL}} \times 100}}{{V_{1} \times 5\;{\text{mL}} \times weight\; of\;sample}} $$

### Lycopene content determination

For lycopene determination, 5 mL sample was diluted with 5 mL of distilled water and agitated in a water bath at 25 °C for 1 h, after which 8.0 mL of solvent (hexane: ethanol: acetone; 2:1:1 ^v^/_v_) was added. The sample solution was covered and vortexed for some minutes, after which it was incubated in the dark for 25 min. Following incubation, 1.0 mL of distilled water was added to the samples and vortexed. The sample solution was then allowed to stand for 10 min and absorbance reading taken using a spectrophotometer at wavelength of 503 nm^26^.

Values were calculated as:

$$ Lycopene\left( {{\text{mg}}\;{\text{mL}}^{ - 1} } \right) = A _{503 } \times 137.4 $$

### Polyphenoloxidase assay (Catechol assay)

Polyphenol oxidase was determined spectrophotometrically by a change in colour of catechol from a colorless to colored benzoquinone solution^27^. Five milliliter (5 mL) sample was diluted with equivalent volume of water and transferred into a test tube and allowed to sediment and the supernatant carefully decanted. Two cubic centimeter (2 cm^3^) of phosphate buffer (pH 7) and 0.1% catechol solution was added to 0.1 cm^3^ of enzyme extract in a test tube. Sample readings were taken with a spectrophotometer (420 nm) previously zeroed with 4 cm^3^ of distilled water and 0.1 cm^3^ of enzyme extract.

### Antifungal activity evaluation

Following storage at room temperature (25 ± 2 °C) for six days, incidence of fungal infection was estimated as the percentage of decayed fruit while disease severity was determined as the diameter of the lesion (mm)^22^. In addition, aerobic total fungal count for tomato fruits in each group was carried out on potato dextrose agar medium using the standard pour plating technique. Tomato fruits in each treatment replicate were crushed into paste and thoroughly mixed together. For antifungal activity estimation, 1 g of crushed tomato from each of the respective fruits was placed aseptically in test tube containing 10 mL of sterile distilled water and vortexed to homogenize. One millilitre of sample was further removed to carry out series of tenfold serial dilutions in test tubes containing 9 mL of sterile distilled water. Pour plating in SDA plates was carried out using known dilutions of the respective treatments, while ensuring the maintenance of aseptic conditions. The media was mixed thoroughly with the sample and aseptically transferred on to petri dish. The plates were incubated in an inverted position at 25 °C for 48 h. Treatment was performed in five replicates while counts were in triplicates.

### Statistical analysis

All experiments were done in triplicate, results are reported as mean ± SD. Data were analysed with the aid of ANOVA using a completely randomized factorial experimental design, with two factors (treatment groups and storage time) while means comparison was carried out using Turkey multiple range test (*P* < 0.05). GraphPad prism 8.0 software (GraphPad Software Inc., San Diego, California) was utilized in drawing charts.

## Results

### Phytochemical composition

The HPLC fingerprint of APPE revealed the presence of quercetin (41.58 ppm), kampferol (1.23 ppm) and a negligible amount of *p*-coumaric acid (8.80 × 10^–2^ ppm) (Supplementary File A).

### SEM analysis

**Figure 1:**
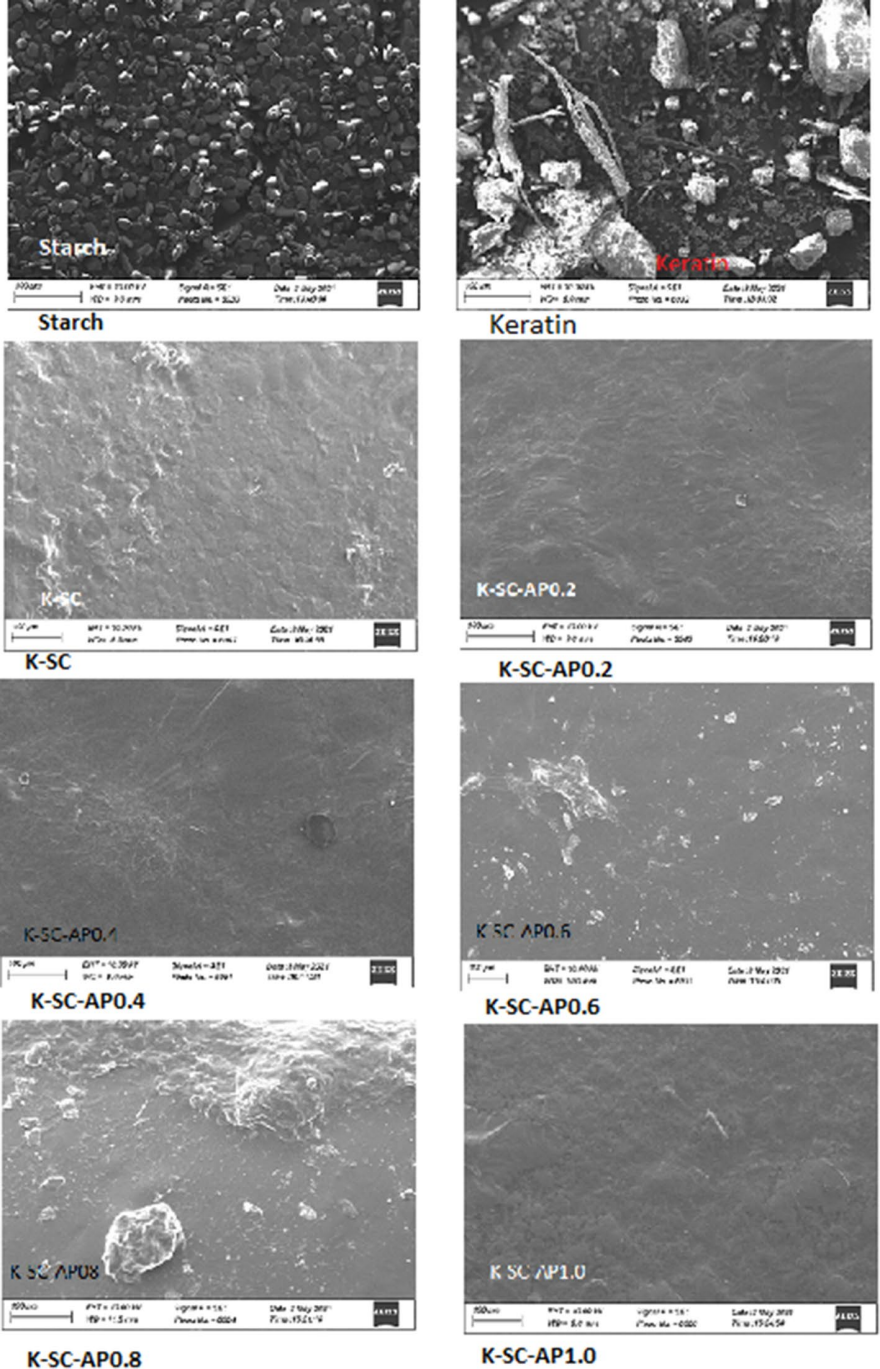
SEM images of keratin-starch films functionalized with avocado pear peel polyphenolic peel extract*. Note* SC, starch; K, keratin; K-SC, keratin-starch composite; while K-SC-AP_0.2_, K-SC-AP_0.4_, K-SC-AP_0.6_, K-SC-AP_0.8_ and K-SC-AP_1.0,_ keratin-starch composite enriched with 0.2 mL, 0.4 mL, 0.6 mL, 0.8 mL and 1.0 mL avocado pear peel polyphenolic extract, respectively.

Surface morphology and microstructure of keratin-starch biofilm with or without APE as revealed by SEM analysis is shown in Fig. 1. Free starch nanoparticles showed uniformly smooth spherically shaped crystals while keratin particles appeared as rod-like fibers. Free starch crystals as were as keratin fibers were not visibly seen in the keratin-starch composites with or without APE. The scanning electron micrographs of the films showed a consistent increasing degree of smoothness with increasing concentration of avocado pear peel polyphenolic extract. Film smoothness showed a high degree of homogeneity of the composite materials as well as encapsulation efficiency of APE in the keratin-starch composite film.

### EDX (elemental profile) analysis

EDX analysis was undertaken to ascertain the formation of keratin-starch composite. Different areas of the film were focused for EDX measurement and the corresponding peaks obtained are shown in Supplementary File B. Elemental composition of keratin, ginger starch, keratin-starch composite film and keratin-starch composite films functionalized with avocado pear peel polyphenolic peel extract, as revealed by EDX spectra showed C, N, O and S as the major atoms in the composite films (Table 2).

**Table 2:** Elemental composition of the fabricated bio-composite film as revealed by EDX analysis.

Element	Atomic weight (%)							
	SC	K	K-SC	K-SC-AP_0.2_	K-SC-AP_0.4_	K-SC-AP_0.6_	K-SC-AP_0.8_	K-SC-AP_1.0_
C K	48.37	51.85	69.33	79.52	88.92	69.95	90.25	74.89
N K	35.51	35.33	12.76	9.32	6.13	18.38	4.83	11.08
O K	0.55	0.1	9.69	4.37	0.61	4.86	0.27	5.02
Al K	0.14	–	0.41	0.35	0.25	0.3	0.11	0.5
S K	7.52	7	6.92	5.59	3.74	5.35	4.07	7.67
Cl K	6.43	5.72						
K K	0.69							
Ca K	0.79							
Si K			0.89	0.74	0.36	0.92	0.47	0.63
Na K				0.02		0.14		0.21
Mg K				0.08		0.11		

*SC* starch, *K* keratin, *K-SC* keratin-starch composite, while K-SC-AP_0.2_, K-SC-AP_0.4_, K-SC-AP_0.6_, K-SC-AP_0.8_ and K-SC-AP_1.0_, keratin-starch composite enriched with 0.2 mL, 0.4 mL, 0.6 mL, 0.8 mL and 1.0 mL avocado pear peel polyphenolic extract, respectively.

### FTIR analysis

**Figure 2:**
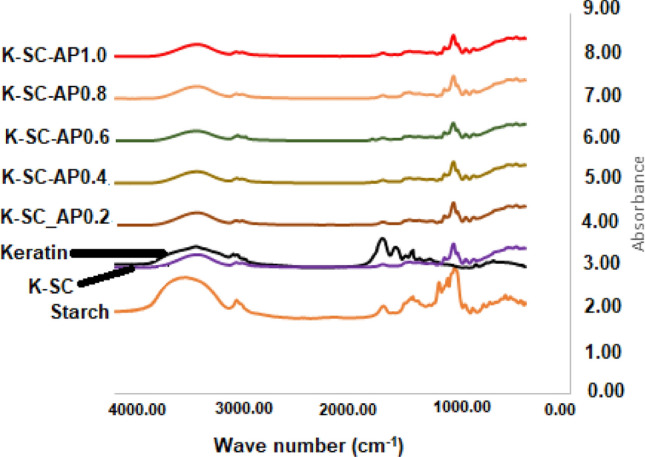
FTIR images of keratin-starch films functionalized with avocado pear peel *polyphenolic* extract*. Note* K-SC, keratin-starch composite; while K-SC-AP_0.2_, K-SC-AP_0.4_, K-SC-AP_0.6_, K-SC-AP_0.8_ and K-SC-AP_1.0,_ keratin-starch composite enriched with 0.2 mL, 0.4 mL, 0.6 mL, 0.8 mL and 1.0 mL avocado pear peel polyphenolic extract, respectively.

Infrared spectra of keratin, ginger starch, and the keratin-starch composites functionalized with avocado pear peel polyphenolic extract are presented in Fig. 2. The FTIR showed a common OH stretching at 3270–3280 cm^−1^ in all the spectra; C–H stretching at 2922–2965 cm^−1^, aliphatic primary C–O stretching at 1647 cm^−1^, in addition, the presence of secondary amide stretching at 1537 cm^−1^ in keratin.

### Transparency test

Film opacity was observed to be significantly (*p* < 0.05) lower in the avocado pear peel enriched keratin-starch film compared to keratin-starch only film. Keratin-starch film containing 0.6, 0.8 and 1.0 mL avocado pear peel polyphenolic extract exhibited 57.3% opacity compared to 26.3% observed for keratin-starch (K-SC).

### Water solubility, pH and weight loss

**Figure 3:**
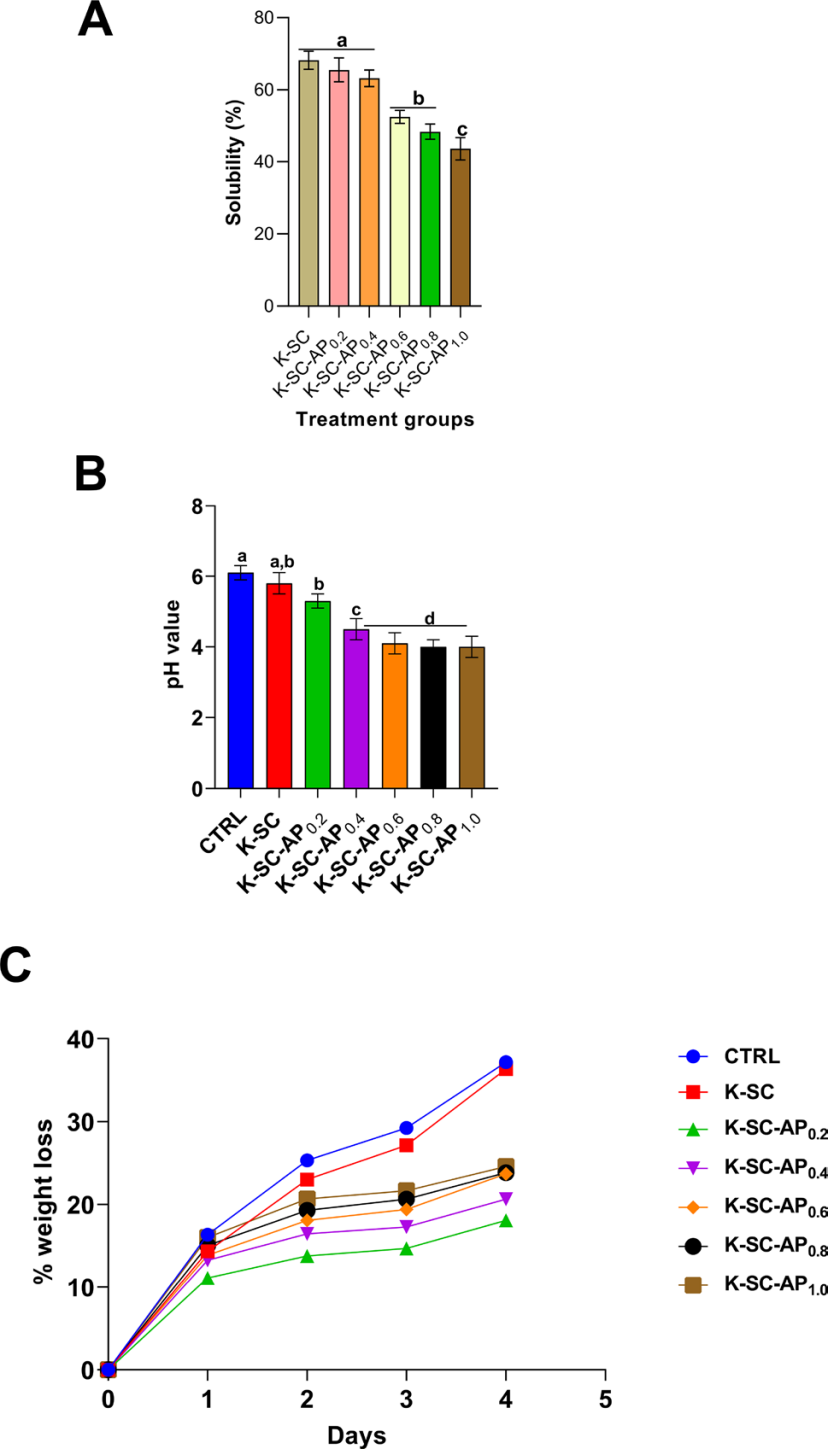
Solubility (**A**), pH (**B**) and percentage weight loss (**C**) of keratin-starch composite containing different concentration of avocado pear peel polyphenolic extract in water. Results are mean ± SEM of triplicate determinations. Bars carrying alphabet is significantly (*p* < 0.05) different from control. *Note* CTRL, control, K-SC, keratin-starch composite coated; K-SC-0.2, K-SC-0.4, K-SC-0.6, K-SC-0.8, and K-SC-1.0, keratin-starch composite enriched with 0.2, 0.4, 0.6, 0.8, and 1.0 mL avocado pear peel polyphenolic extracted coated tomato, respectively.

The solubility of the fabricated film was observed to decrease with increasing concentration of avocado pear peel polyphenolic extract. However, no significant (*p* > 0.05) difference was observed in the rate of solubility between films containing the varying concentration of the extract (Fig. 3A). Tomato fruits coated with avocado pear peel polyphenolic extract-enriched keratin-starch film had significantly (*p* < 0.05) lower pH values compared control. Tomato fruits coated with 0.6, 0.8 and 1.0 mL avocado pear peel polyphenolic extract-enriched film had statistically similar pH value (Fig. 3B). The loss in weight in the different treatment groups is shown in Fig. 3C. Tomato fruits coated with distilled water (control) and those coated with keratin-starch film without extract had significantly (*p* < 0.05) higher loss in weight compared to those coated with films containing varying concentrations of the extract. Weight loss in tomato fruits coated with extract containing films was observed decrease with increasing concentration of the extract.

### Titratable acidity, ascorbic acid and lycopene content

**Figure 4:**
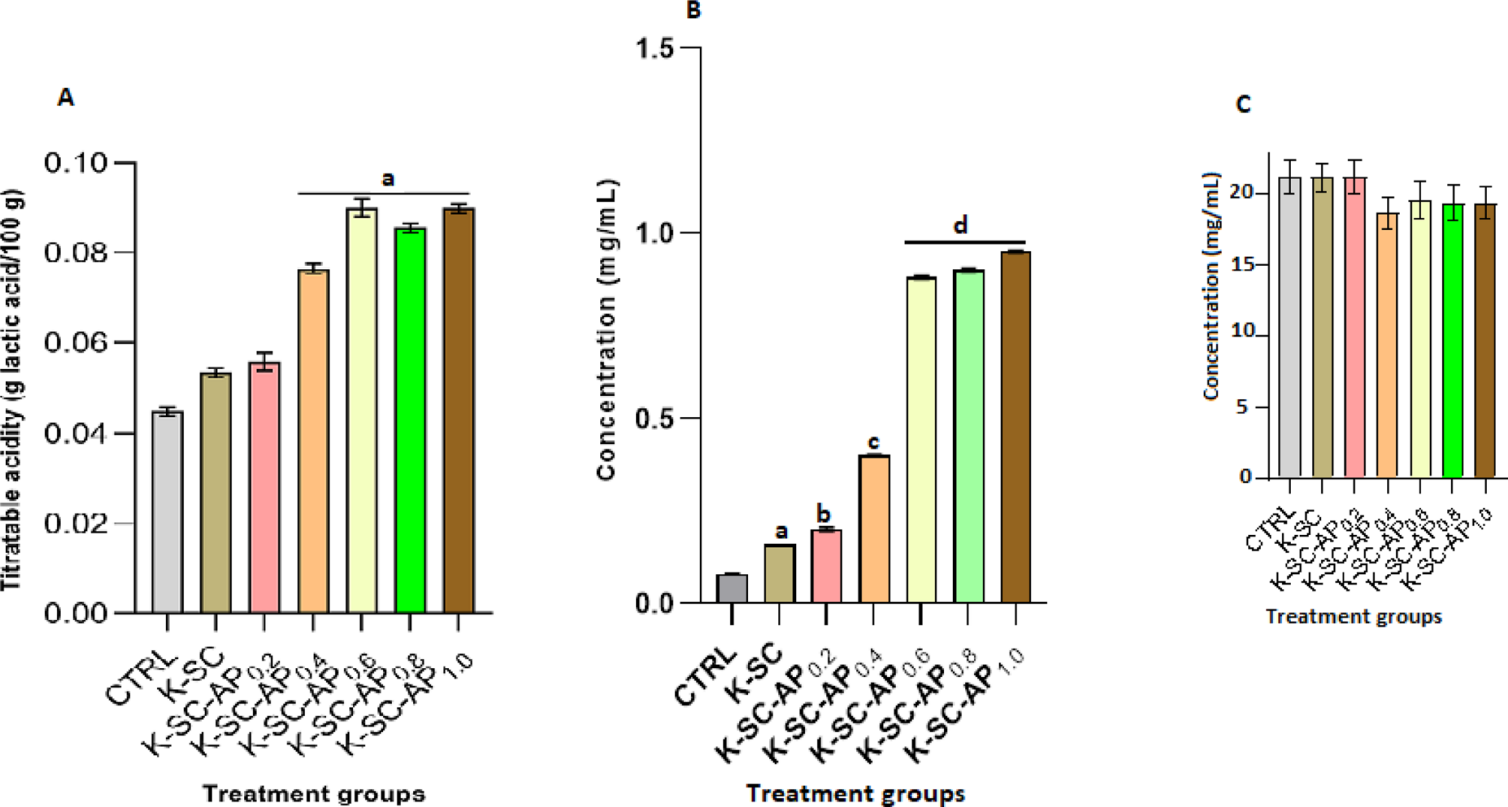
Titratable acidity (**A**), ascorbic acid concentration (**B**) and lycopene content (**C**) of fungi-infected tomato fruits coated with avocado pear peel polyphenolic extract-based keratin-starch composite film for a period of six days at room temperature (25 ± 2 °C). Results are mean ± SEM of triplicate determinations. Bars carrying alphabet is significantly (*p* < 0.05) different from control. *Note*: CTRL, control, K-SC, keratin-starch composite coated; K-SC-0.2, K-SC-0.4, K-SC-0.6, K-SC-0.8, and K-SC-1.0, keratin-starch composite enriched with 0.2, 0.4, 0.6, 0.8, and 1.0 mL avocado pear peel polyphenolic extracted coated tomato, respectively.

Titratable acid levels in tomato fruits subjected to the different treatment groups is presented in Fig. 4A. Tomato fruits coated with avocado pear peel polyphenolic extract-containing film had significantly (*p* < 0.05) higher titratable acid level compared to tomato fruits coated with keratin-starch only and distilled water (control). The ascorbic acid concentration in the different treatment groups is shown in Fig. 4B. Tomato fruits coated with distilled water (control) and those coated with keratin-starch film without extract had significantly (*p* < 0.05) lower ascorbic acid level compared to those coated with films containing varying concentrations of the extract. Ascorbic acid concentration in tomato fruits coated with extract containing films was observed to increase with increasing concentration of avocado pear peel polyphenolic extract in the film. No significant statistical difference was observed in lycopene content between infected tomato fruits coated with distilled water (control) and those coated with avocado pear peel polyphenolic extract-based keratin-starch composite film (Fig. 4C).

### Catechol oxidase and antifungal activities

**Figure 5:**
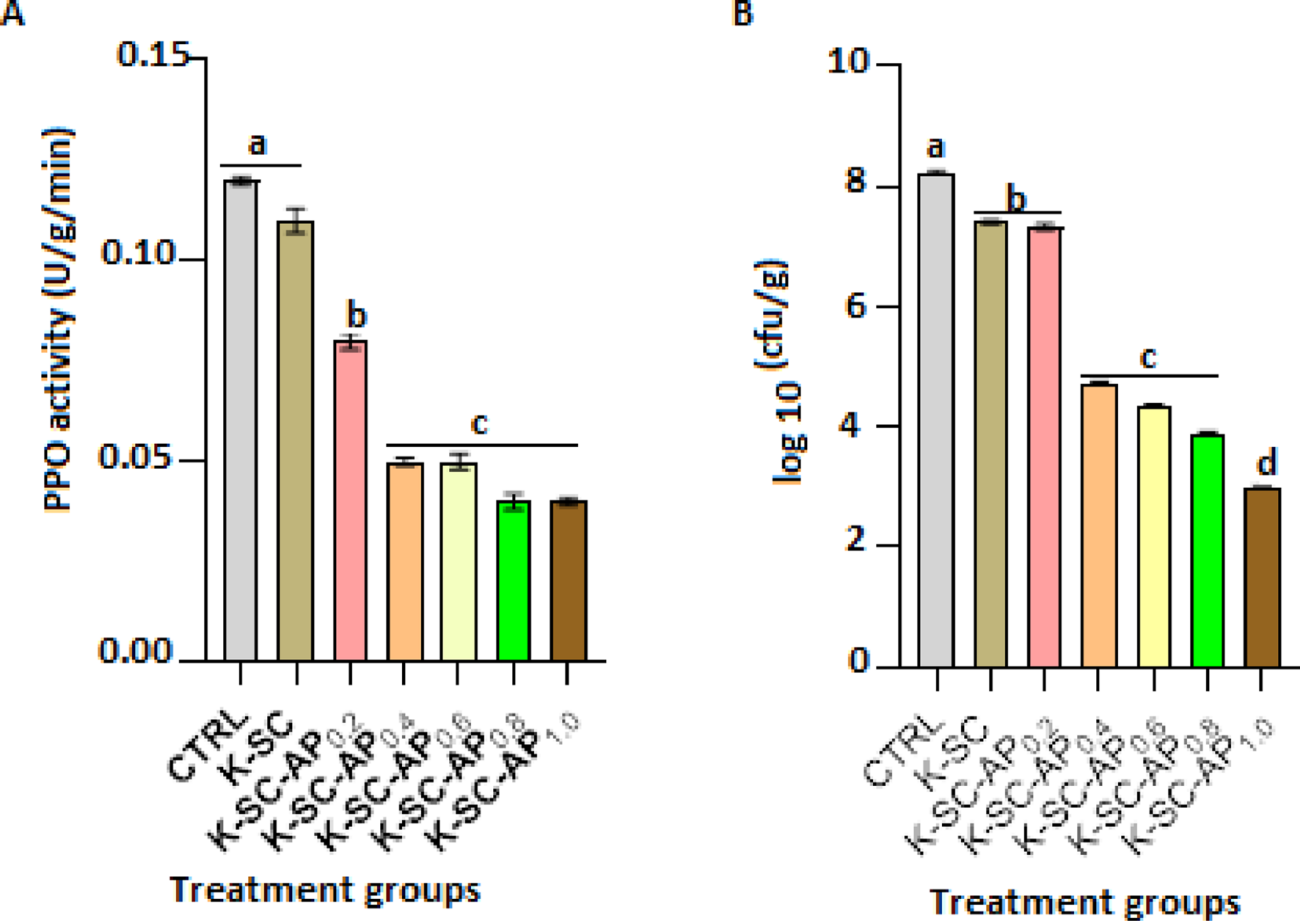
Catechol oxidase activity (**A**) and antifungal activity (**B**) of fungi-infected tomato fruits coated with avocado pear peel polyphenolic extract-based keratin-starch composite film for six days at room temperature (25 ± 2 °C). Results are mean ± SEM of triplicate determinations. Bars carrying alphabet are significantly (*p* < 0.05) different from control. *Note* CTRL, control, K-SC, keratin-starch composite coated; K-SC-0.2, K-SC-0.4, K-SC-0.6, K-SC-0.8, and K-SC-1.0, keratin-starch composite enriched with 0.2, 0.4, 0.6, 0.8, and 1.0 mL avocado pear peel polyphenolic extracted coated tomato, respectively.

Infected tomato fruits coated with avocado pear peel polyphenolic extract-based keratin-starch composite film exhibited significantly (*p* < 0.05) lower catechol oxidase activity, when compared with tomato fruits coated with keratin-starch composite alone and those coated with distilled water. Furthermore, the enzyme activity, though not significantly changed in tomato fruits coated with keratin-starch composite films containing 0.4, 0.6, 0.8 and 1.0 mL avocado pear peel extract, was significantly lower compared to those fruits coated with 0.2 mL avocado pear peel polyphenolic extract-based composite film (Fig. 5A). Incidence and severity of *A. flavus* and *A. niger* infection were significantly reduced in tomato fruits coated with keratin-starch films functionalized with avocado pear peel extract (Table 3) compared to control. Coatings containing 0.8 and 1.0 mL avocado pear peel polyphenolic extract exhibited the most significant protective effect compared to tomato fruits coated with film containing lower concentrations of the extract. Tomato fruits coated with both keratin-starch composite only and those coated with avocado pear peel polyphenolic extract-based composite film had significantly (*p* < 0.05) lower fungal loads, when compared with control. Keratin-starch composite containing 1.0 mL avocado pear peel extract exhibited the best antifungal activity, when compared with composite containing lower concentration of the extract (Fig. 5B).

**Table 3:** Disease severity (mm) and incidence (%) of fungal spoilage on tomato fruits artificially inoculated with *A. flavus* and *A. niger* spores and coated with keratin-starch (K-SC) film functionalized with avocado pear peel polyphenolic extract.

	Control	Treatment groups					
		K-SC	K-SC-AP_0.2_	K-SC-AP_0.4_	K-SC-AP_0.6_	K-SC-AP_0.8_	K-SC-AP_1.0_
Disease incidence (%)	71.8^d^	53.5^c^	48.7^b,c^	43.5^b^	41.2^b^	35.3^a,b^	33.9^a^
Disease severity (mm)	21.1^c^	16.5^b^	15.7^b^	14.8^a,b^	13.5^a^	13.1^a^	12.8^a^

Control, infected tomato fruits coated with distilled water; K-SC, infected tomato fruits coated with keratin-starch film; while K-SC-AP_0.2_, K-SC-AP_0.4_, K-SC-AP_0.6_, K-SC-AP_0.8_ and K-SC-AP_1.0,_ infected tomato fruits coated with keratin-starch film functionalized with 0.2 mL, 0.4 mL, 0.6 mL, 0.8 mL and 1.0 mL avocado pear peel polyphenolic extract, respectively. Data on the same row carrying different alphabets are statistically significant (*p* < 0.05).

## Discussion

Climacteric, fruits such as tomato constitute a good system for evaluating metabolic changes involved in the maturation and ripening of fruits. Several factors, including physicochemical changes, such as weight loss, pH, as well as transpiration, pathogen infestation, etc. contribute to postharvest losses during storage^28,29^. Efforts aimed at regulating these factors among other things are strategic in enhancing storage life as well as postharvest losses of agricultural products.

The degree of smoothness or homogeneity of the film fabricated using ginger starch, chicken feather keratin and avocado pear peel extract as revealed is indicative of a high rate of dispersion or miscibility of the individual components of the film resulting in no crack or cavity within the film. It also attested to the level of cross linking or intermolecular associations that could have resulted from the amide nitrogen and oxygen with the hydroxyl groups present in both starch and the phenolic rings of the extract. The reduction in film transparency due to the incorporation of avocado pear peel polyphenolic in the film in this study is advantageous in its application as an edible coating. Film opacity could aid in the reduction in light intensity or penetration into the coated tomatoes thus preventing the potential oxidation of molecules such as ascorbic acid and phenolics antioxidant present in the tomato fruits. Oluba et al.^12^ recently demonstrated biocomposites film containing keratin exhibited a reduction in film transparency.

In this study, weight loss was observed to decrease with increasing concentration of avocado pear polyphenolic extract in the keratin-starch film. Keratin-starch film coating alone had no appreciable effect in preserving the weight loss. It could thus be argued that the film only served as a carrier for the extract. One of the greatest challenges in the transportation and storage of fruits is the unregulated movement of water. The loss or gain of water during storage is undesirable. The shrinkage of fruits due to excessive water loss most often results to loss in economic value of fruits. In addition, excessive loss of water measured as weight loss in this study could affect the juiciness of fruits^30^.

Generally, the titratable acidity of tomato fruits used in the study was slightly below 0.1 μeq g^−1^. This is low compared to the value reported by Sree et al*.*^31^ for chitosan coated tomato fruit coated for a period of 30 days. This is not unlikely because this study unlike Sree et al.^31^ utilized already ripened tomato fruits with low level of metabolic activity. The rate of respiration in ripened tomatoes is low compared to green tomatoes with higher rate of respiration. Low level of titratable acidity as measured in this study signifies low concentration of citric acid being the most abundant organic acid in tomato fruits. It has been established that there is a concomitant loss of citric acid concentration with increasing maturity and ripening^32^. The ripening process in tomato fruit is accompanied by increased metabolic breakdown of citric acid to sugars. In addition, there is a further loss of acid as a consequence of high rate of ethylene production as well as increased respiratory rate during ripening^33^. The concomitant higher concentration of ascorbic acid recorded in the avocado pear peel polyphenolic extract enriched keratin-starch film coated tomato fruits as opposed to uncoated tomato fruits in this study could be a attributable to reduce metabolic conversion of ascorbic acid to sugars as well as low respiratory rate. This observation further reinforce the potential ability of the keratin-starch-avocado peel polyphenolic extract enriched film to serve as a barrier to oxygen.

The discolouration of fruits, which most often results in consumer rejection, due to unacceptable aesthetic appearance is a major challenge in fruit production. Polyphenol oxidase activity as observed in this study was not significantly different between coated and uncoated fruits. This is significant because polyphenol oxidase catalyzes the process of enzymatic browning of fruits which results in fruit discolouration. The tomato fruits used in this study were reddish and fully ripened hence no appreciable browning reaction is expected at that stage. Catechol oxidase activity was observed to be lower in tomato fruits coated with avocado pear peel polyphenolic extract enriched keratin-starch film in comparison to uncoated fruit in this study. In addition, catechol oxidase activity showed positive correlation with ascorbic acid level across the treatment groups. High ascorbic acid concentration of the avocado pear peel polyphenolic extract enriched keratin-starch film coated tomato lowers the pH level in these tomato fruits. Catechol oxidase has been shown to be catalytically active between pH 4–pH 8. Low pH due to high ascorbic acid content could interfere with the binding of the enzyme to its active site copper^34^. Enzymatic browning of fruits not only affects the appearance of fruits but also their nutritional Quality. Over fifty percent of fruit losses due to tropical fruits are attributable to enzymatic browning^35^.

The concurrent reduction in disease incidence and severity due to application of avocado pear peel polyphenolic extract functionalized keratin-starch coating in this study suggests that the antifungal activity of the coating could be fungistatic and not fungicidal as the coating does not completely hinder the growth of the pathogen. This observation is in agreement with the report of Fagundes et al.^22^ as well as Palou et al.^36^. Results from this study evidently showed that avocado pear peel polyphenolic extract remarkably enhanced the antimicrobial activity of keratin-starch composite film, when applied as coating on infected tomato fruits. This observation is corroborated by reports from earlier studies^37,38^, demonstrating that the incorporation of plant extract into edible coatings significantly improves their antimicrobial activity. Kubheka et al*.*^38^ demonstrated that the incorporation of *Moringa oleofera* leaf extract remarkably enhanced the antifungal activity of edible coating against *Colletotrichum gloeosporioides* infection on Maluma avocado fruits in vitro. Similarly, Tesfaye et al.^39^ reported the inhibition of *C. gloeosporioides* growth in vitro by moringa leaf extract. Recently, Yusoff et al.^40^ demonstrated the inhibition of gray mold disease in tomato fruit by the application of *Vernonia amygdalina* leaf extract-based emulsion. The antimicrobial action of plant extract has been attributed to the diversity of phytochemicals or secondary metabolites present in them. The fruits, leaves, seeds, roots and bark of plant are rich sources of secondary metabolites with proven antimicrobial activity. The HPLC fingerprint obtained for avocado pear peel used in this study showed the presence of flavonoids and phenolic compounds. Specifically, quercetin and kampferol were identified as the major phenolics present in avocado pear peel extract. In addition, it is pertinent to know that the lowering effect of the avocado peel enriched keratin-starch film on the pH as well its potential effect on the retention of ascorbic acid content of the infected tomato fruits may have played significant role in its antifungal action as demonstrated in this study. The high level of citric acid vis-à-vis the low pH could constitute and unfavourable environment for fungal growth.

## Conclusion

The results presented in this study demonstrated that the application of an avocado pear peel polyphenolic extract activated keratin-starch coating on fungal-infected tomato fruit remarkably inhibited the incidence of infection as well as the severity of infection and in addition conserved their organoleptic and nutritional qualities. Thus demonstrating the antimicrobial activity of avocado peel polyphenolic extract as well as the potential application of keratin-starch composite film in food packaging and post-harvesting storage of tomato fruits. However, further research on the water vapour and oxygen/carbondioxide permeability as well as sensory evaluation of the coated tomato fruits are required to further justify its application as an edible coating.

## Supplementary Information

Supplementary Information.
