# Supplementary material for: Fabrication, characterization and antifungal evaluation of polyphenolic extract activated keratin starch coating on infected tomato fruits
Source: Sci Rep. 2022 Mar 14;12:4340. doi: 10.1038/s41598-022-07972-0 (PMC8921230; doi:10.1038/s41598-022-07972-0)
Supplement: Supplementary file 1 — Supplementary Information. [file 41598_2022_7972_MOESM1_ESM.docx]

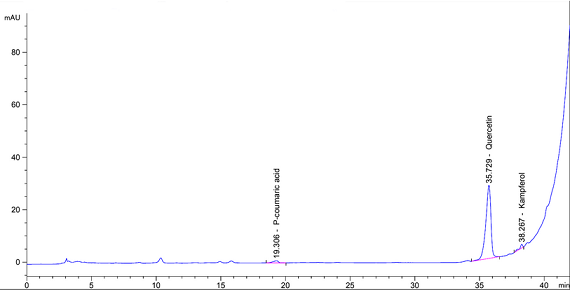


Supplementary A: HPLC fingerprint of avocado pear peel polyphenolic extract


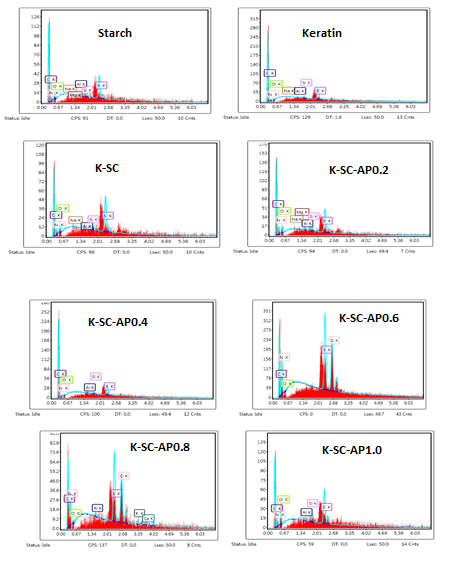


Supplementary file B: EDX images of keratin-starch films functionalized with avocado pear peel polyphenolic peel extract*.* Note: K-SC, keratin-starch composite; while K-SC-AP_0.2_, K-SC-AP_0.4_, K-SC-AP_0.6_, K-SC-AP_0.8_ and K-SC-AP_1.0,_ keratin-starch composite enriched with 0.2 mL, 0.4 mL, 0.6 mL, 0.8 mL and 1.0 mL avocado pear peel polyphenolic extract, respectively.
